# The Influence of Sleep over Disease

**Published:** 1862-06

**Authors:** 


The Influence of Sleep over Disease.—In all forms and conditions of disease, both acute and chronic, the state of the patient as to *sleep* is an important consideration, both as regards his comfort, and also as regards the satisfactory progress of his case. The nature of this condition of animal life we do not fully understand ; we only know that it is a necessary one, and having a vast influence on the state of the system. Its purpose seems to be to afford an opportunity, by the suspension of certain activities of the system which require the exhaustion of those powers that emanate from the nervous system, for the reinforcement of those powers. *It is also*

*during sleep that the repair of the tissues by nutrition is provided for.* Not that all nutrition is suspended during our waking hours, or that all waste is suspended during sleep; but that in two states of sleeping and waking there is respectively a large predominance of the repair and the waste.

Sleep is not merely rest, as it has been sometimes considered, an entire rest of all the organs at once; it is something specifically different. It is a condition of an entirely different nature, and a condition for which rest is not, in any sense, a substitute. The mere facts of existence, without exercise, without fatigue; the simple going on of life—implies a certain expenditure of force, which renders necessary, at certain intervals, a suspension of those functions of the brain and nervous system which are subservient to the phenomena of mind. It is possible that ordinary rest might afford an opportunity for the nutrition of all these tissues, except those which are the agents of the mind. But it seems to be necessary, for the repair of these, that the functions of the mind should also be suspended. Of the physical condition of the brain in sleep, and also concerning the peculiar state of the mind in sleep, notwithstanding the many theories which have been formed concerning them, we know nothing with certainty; and this is not necessary to the practical management of the sick. What should guide us is the knowledge that a certain amount of sleep, at proper intervals, is an absolute necessity, and that its absence or its deficiency is always a great evil, and to be prevented by every possible means. In acute diseases a sufficient amount of quiet sleep is at once a favorable indication of the nature and issue of a case, and also is an important agent in the promotion of a favorable issue. Its absence, on the contrary, is, *pro tanto*, an unfavorable indication as to the result, and also promotes an unfavorable issue. Want of sleep adds to the sufferings of the patient, and also to his exhaustion, and consequently interferes with the success of the sanitary process, and impairs the power of recovery. In every point of view, then, the state of the patient in this respect becomes the object of special attention. Salutary changes in the condition of a patient will be often found to take place during sleep, and to manifest themselves most obviously on awaking from that which has been sound and refreshing.—*Medical and Surgical Reporter.*

